# Interaction of historical and modern Sardinian African swine fever viruses with porcine and wild-boar monocytes and monocyte-derived macrophages

**DOI:** 10.1007/s00705-018-04140-6

**Published:** 2019-01-10

**Authors:** Silvia Dei Giudici, Giulia Franzoni, Piero Bonelli, Donatella Bacciu, Giovanna Sanna, Pier Paolo Angioi, Mauro Ledda, Giovannantonio Pilo, Paola Nicolussi, Annalisa Oggiano

**Affiliations:** 1 Istituto Zooprofilattico Sperimentale della Sardegna, Via Vienna 2, 07100 Sassari, Italy; 2 Department of Veterinary Medicine, University of Sassari, Via Vienna 2, 07100 Sassari, Italy

## Abstract

African swine fever (ASF) is a contagious viral disease of wild and domestic pigs that is present in many parts of Africa, Asia and Europe, including Sardinia (Italy). Deletions in the EP402R and B602L genes have been found in almost all ASF virus (ASFV) strains circulating in Sardinia from 1990 onwards, and modern Sardinian strains (isolated after 1990) might have acquired some selective advantage compared to historical ones (isolated before 1990). Here, we analysed the host cell responses of wild boars and domestic pigs upon infection with virus variants. Higher intracellular levels of the late protein p72 were detected after infection with the modern strain 22653/14 compared to the historical strain Nu81.2, although both isolates grew at the same rate in both monocytes and monocyte-derived macrophages. Higher cytokine levels in the supernatants of ASFV-infected pig monocytes compared to pig macrophages and wild-boar cells were detected, with no differences between isolates.

## Introduction

African swine fever (ASF) is a contagious and often fatal viral disease of domestic pigs and wild boar [22] that is currently endemic in many sub-Saharan African countries, the Russian Federation, Transcaucasia, some East European countries, and Sardinia [1]. A recent ASF outbreak was also reported in China [8]. There is still neither a licensed vaccine nor a treatment available, and disease-control measures rely on stamping out and movement restrictions, resulting in extreme losses for producers [22]. In Sardinia, the disease first occurred in 1978, and despite many eradication programmes it is still endemic [3, 5, 18]. So far, all Sardinian ASFV isolates have been found to belong to p72 (B464L) genotype I, whereas genotype II ASFV isolates are circulating in the other European countries, Transcaucasia, Russia, and China, [1, 8]. The epizootic cycle of ASFV in Sardinia is characterized by the absence of Ornithodoros ticks [18], which are biological vectors for ASFV and have been implicated in the long-term maintenance of the virus in Spain and Portugal (*O. erraticus*) and East and South Africa (*O. moubata*) [1]. There is instead evidence of endemically infected wild boar populations [5, 16, 18]. The role of wild boars in ASFV epidemiology in Sardinia remains controversial, but several authors agree on their secondary role in ASF transmission and instead emphasize the importance of the interaction between free-ranging pigs and wild boars for the persistence of the disease [13, 18]. Previous studies have shown that ASFV in wild boars in Sardinia tends to be self-limiting in the absence of contacts with free-ranging pig populations [14, 20].

Molecular characterization of Sardinian ASFV strains isolated from domestic and wild pigs showed high genetic similarity. Indeed, an analysis of the genes coding for p72 and p54 proteins showed that all Sardinian strains can be classified within genotypes I and Ia, respectively [9, 23]. Differences were instead observed in the B602L gene, which is involved in viral morphogenesis [4], allowing the differentiation of Sardinian isolates in two temporally related subgroups (X and III). Almost all of the strains isolated from 1990 onwards (subgroup X) showed the deletion of 12-13 tetramers [9] with respect to those isolated before 1990 (subgroup III). Likewise, [23] reported an identical temporal subdivision of Sardinian viruses into two subgroups differing from the deletion of a six-amino-acid repeat at the C-terminus of the CD2v protein encoded by the EP402R gene, which is characteristic of the strains isolated after 1990. Ultimately, almost all of the Sardinian ASF viruses isolated after 1990 (modern strains) showed deletions in both the B602L and EP402R genes if compared to viruses isolated before 1990 (historical strains). The modern strains may have acquired some selective advantage, as suggested by their rapid and almost complete displacement of the historical strains.

We performed an *in vitro* study to better characterise the phenotype of two representative viruses that have circulated in Sardinia since the detection of ASFV on the island: the modern strain 22653/14 and the historical strain Nu81.2. Differences in their ability to infect monocytes and monocyte-derived macrophages (moMΦ) of domestic and wild pigs were assessed. Furthermore, the present study aimed to provide a better understanding of the *in vitro* responses of wild-boar myeloid cells against ASFV. For this purpose, we analysed the susceptibility to infection, growth kinetics, and cytokine responses of both pig and wild-boar monocytes and macrophages against historical and modern Sardinian isolates that differ due to deletions in the EP402R and B602L genes. Despite the need to better understand the epidemiological role of wild boars in the dissemination and persistence of ASFV in Sardinia, to our knowledge, no previous studies have ever compared monocyte/macrophage responses to ASFV between pigs and wild boars.

## Materials and methods

### Animals

Healthy ASFV-naïve crossbred Large White × Landrace pigs and wild boars, 6-24 months of age, were housed at the experimental facilities of IZS della Sardegna (Sassari, Italy) or University of Sassari, Faculty of Veterinary Medicine (Sassari, Italy). Animal husbandry and handling procedures were performed in accordance with the local ethics committee and in agreement with the guide for use of laboratory animals of the Italian Ministry of Health. The ASFV-negative status of the animals was confirmed by three different laboratory tests: PCR, a commercial ELISA test (Ingenasa, Madrid, Spain), and an immunoblotting test, as suggested by the Manual of Diagnostic Tests and Vaccines for Terrestrial Animals [19].

### Viruses

Two virulent haemadsorbing Sardinian field strains were used in this study: the modern strain 22653/14, characterized by a deletion of one of the PPPKPC repeats in the EP402R gene and 13-amino-acid tetramer repeats in the B602L gene, and the historical strain Nu81.2, without deletions in either gene (Exotic Disease Laboratory ASF Virus Archive, IZS). Strains 22653/14 and Nu81.2 were isolated from naturally infected pigs collected during ASF outbreaks in 2014 and 1981, respectively. Sardinian isolates were propagated *in vitro* by inoculation of sub-confluent monolayers of porcine monocytes/macrophages as described previously [15]. Viral titers were obtained by serial dilution of the virus suspension on monocytes/macrophages, followed by hemadsorption [15]. Mock-virus supernatants were prepared in an identical manner from monocyte/macrophage cultures.

### Cells

Porcine monocytes were obtained as described previously [6]. Monocytes were seeded at a concentration of 8-10 × 10^5^ live cells/well in a 12-well plates (Greiner CELLSTAR, Sigma). To differentiate monocytes into monocyte-derived macrophages (moMΦs), cells were cultured for 5 days at 37 °C with 5% CO_2_ in RPMI 1640 medium with 10% foetal bovine serum (FBS) supplemented with 50 ng of recombinant human macrophage colony stimulating factor (M-CSF) (eBioscience, San Diego, USA) per ml [6].

### ASFV infection of monocytes/moMΦs and growth curves

Culture medium from monocytes and moMΦ cultures were removed and replaced with fresh un-supplemented medium containing ASFV strain 22653/14 or Nu81.2 at a multiplicity of infection (MOI) of 1. To evaluate ASFV growth kinetics, these cells were instead infected at an MOI of 0.01 with the modern strain 22653/14 or the historical strain Nu81.2 ASFV. Mock-infected controls were included in each experiment. After 90 minutes of incubation at 37 °C and 5% CO_2,_ the virus inoculum was removed, the cells were washed with unsupplemented RPMI-1640 medium, and fresh monocyte medium was added to the wells. Cells were incubated at 37 °C and harvested at 18 hours postinfection (pi). To evaluate growth kinetics, culture supernatants were instead collected at 0, 24, 48, and 72 hours pi. Viral infection was assessed by evaluation of intracytoplasmic p72 expression by flow cytometry, and culture supernatants were collected to determine viral levels or cytokine release in response to ASFV infection as described previously [7]. Culture supernatants were stored at -80 °C after collection until analyzed.

### Cytofluorimetric analysis

Cytofluorimetric analysis was performed as described previously [6]. In brief, cells were harvested from cultures and transferred to wells of a 96-well round-bottom plate. Viability was assessed by staining the cells using a LIVE/DEAD® Fixable Far Red Dead Cell Stain Kit (Thermo Fisher Scientific) for 30 minutes at 4 °C, and the cells were then washed twice with PBS supplemented with 2% FBS. Cells were fixed and permeabilised using Leucoperm (Bio-Rad, Hercules, USA) according to manufacturer’s instructions. Intracellular levels of the late viral protein p72 were determined using an anti-p72-FITC antibody (18BG3, Ingenasa). Flow cytometry analysis was performed on an FACSCalibur instrument (BD Biosciences), and at least 5000 live monocytes/moMΦs were acquired. Gates for p72 protein were set using the mock-infected controls [6].

### Analysis of the cytokine levels in culture supernatants of monocytes and moMΦs

Measurement of IL-1α, IL-1β, GM-CSF, IL-6, IL-10, IL-12, IL-18 and TNF-α was performed using a Porcine Cytokine/Chemokine Magnetic Bead Panel Quantikine assay (Merck Millipore, Darmstadt, Germania) and a Bioplex MAGPIX Multiplex Reader (Bio-Rad), according to the manufacturers’ instructions.

### Data analysis and statistics

All experiments were performed in triplicate (flow cytometry and ELISA) or duplicate (virus titration) and repeated at least three times with different blood donor pigs or wild boars. Graphical and statistical analysis was performed using GraphPad Prism 8.0 (GraphPad Software Inc, La Jolla, USA) and Minitab (Minitab Inc., Coventry, UK). All data were checked for normality using the Anderson Darling test. Data are presented as mean values with standard deviations (SD) quoted to indicate the uncertainty around the estimate of the group mean. A non-parametric Mann-Whitney test or a Kruskal-Wallis test were used; a *p*-value < 0.05 was considered statistically significant.

## Results and discussion

### Susceptibility of pig and wild-boar monocytes and moMΦs to ASFV infection with Sardinian isolates

**Fig. 1:**
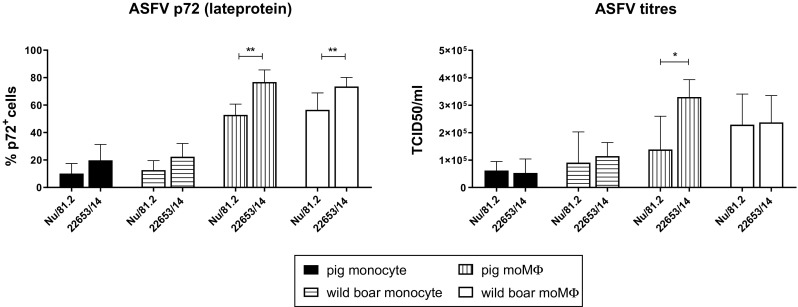
Susceptibility of pig and wild-boar monocytes and monocyte-derived macrophages to infection. Pig and wild-boar blood-derived monocytes were infected immediately or differentiated into macrophages (moMΦs). Monocytes and moMΦs were infected with the virulent historical strain NU81.2 or the modern strain 22653/14 at an MOI of 1, alongside mock-infected controls. At 18 hours pi, the percentage of p72^+^ cells was determined using flow cytometry, and the level of virus in culture supernatants was determined by titration. The mean data +/- SD from three independent experiments utilizing three different pigs and three different wild boars are shown. For each cell type, the values for NU81.2 and 22653/14 were compared using the Mann-Whitney test. ***, *p* < 0.001; **, *p* < 0.01; *, *p* < 0.05

Susceptibility of monocytes and moMΦs to ASFV infection was assessed by quantification of intracytoplasmic p72 expression and viral titers in cell culture supernatants. In accordance with previous studies, porcine macrophages were more susceptible to ASFV infection than monocytes [2, 17, 21], and the same results were observed in wild-boar cells (Fig. 1). The use of hM-CSF has been used previously to differentiate porcine monocytes into macrophages [6, 24] and our results confirm that this protocol is also suitable in wild boars. Our analysis also showed higher levels of p72^+^ cells after infection with the modern strain 22653/14 than after infection with the historical strain Nu81.2 (Fig. 1). Infection with these isolates resulted instead in similar viral levels in culture supernatant; only slightly higher viral levels of 22653/14 compared to Nu81.2 were detected in pig moMΦ supernatants. Furthermore, no differences were observed between domestic pigs and wild boars; both monocytes and macrophages were found to have comparable levels of p72^+^ cells and viral titers, showing similar susceptibility to infection with either modern or historical isolates (Fig. 1).

### Kinetic analysis of ASFV replication in pig and wild-boar monocyte and moMΦs

**Fig. 2:**
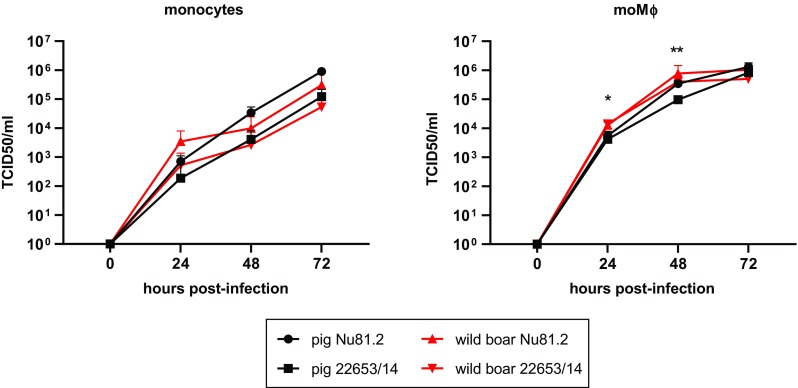
Growth kinetics of Sardinian ASFV strains in pig and wild-boar monocytes and monocyte-derived macrophages. Pig and wild-boar blood-derived monocytes were infected immediately or differentiated into macrophages (moMΦs). Monocytes and moMΦs were then infected with the virulent historical strain Nu81.2 or the modern strain 22653/14 at an MOI of 0.01. At 0, 24, 48, and 72 hours pi, duplicate samples were collected, and virus levels in culture supernatants were determined by titration. The mean data +/- SD from three independent experiments utilizing three different pigs and three different wild boars are shown. For each cell type, at each time pi, the values for Nu81.2 and 22653/14 were compared using the Mann-Whitney test. ***, *p* < 0.001; **, *p* < 0.01; *, *p* < 0.05

Next, a kinetic analysis of the infection of pig and wild-boar monocytes and moMΦs with these two Sardinian ASFV isolates was performed (Fig. 2). An MOI of 0.01 was used, and replication was measured by determining viral titers in cell culture supernatants overtime. This analysis showed that both viruses replicated efficiently in either monocytes or moMΦs, with no statistically significant differences between the historical strain Nu81.2 and the modern strain 22653/14 (Fig. 2). Only minor differences were observed between pig and wild-boar moMΦs : slightly higher viral levels in culture supernatants of ASFV-infected wild-boar moMΦs compared to pig moMΦs infected with the modern 22653/14 but not the historical Nu81.2 were detected at 24 and 48 hours pi (Fig. 2). Our results might suggest that 22653/14 has less ability to replicate in pig moMΦs, but those differences were not observed at later time points (72 hours pi), and experiments should be carried out on a larger number of animals to confirm this finding.

### Cytokine responses of ASFV-infected pig and wild-boar monocytes and moMΦs

Finally, the cytokine responses of monocytes and monocyte-derived macrophages to both strains were assessed. The levels of eight different cytokines were tested, and we found that monocytes released higher levels of cytokines after ASFV infection than did macrophages, in accordance with our previous study [6].

**Fig. 3:**
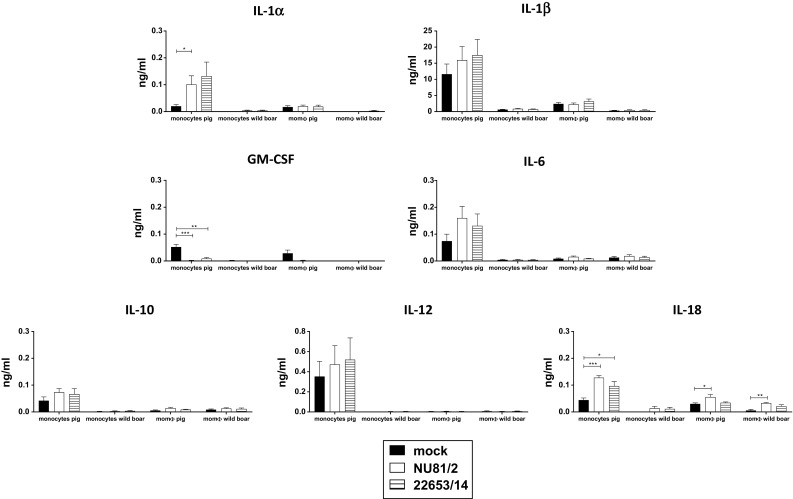
Investigation of cytokines release by monocytes and moMΦs in response to ASFV infection. Pig and wild-boar blood-derived monocytes were infected immediately or differentiated into macrophages (moMΦs). Monocytes and moMΦs were infected with the virulent historical strain NU81.2 or the modern strain 22653/14 at an MOI of 1, alongside mock-infected controls. At 18 hours pi, culture supernatants were collected, and the concentrations (mg/ml) of GM-CSF, IL-1α, IL-1β, IL-6, IL-10, IL-12, and IL-18 were determined. The mean data +/- SD from three independent experiments utilizing three different pigs and three different wild boars are shown. For each cytokine, the values obtained using ASFV-infected monocytes or moMΦs were compared to those from the mock-infected control, using a one-way ANOVA followed by the Kruskal-Wallis test. ***, *p* < 0.001; **, *p* < 0.01; *, *p* < 0.05

**Fig. 4:**
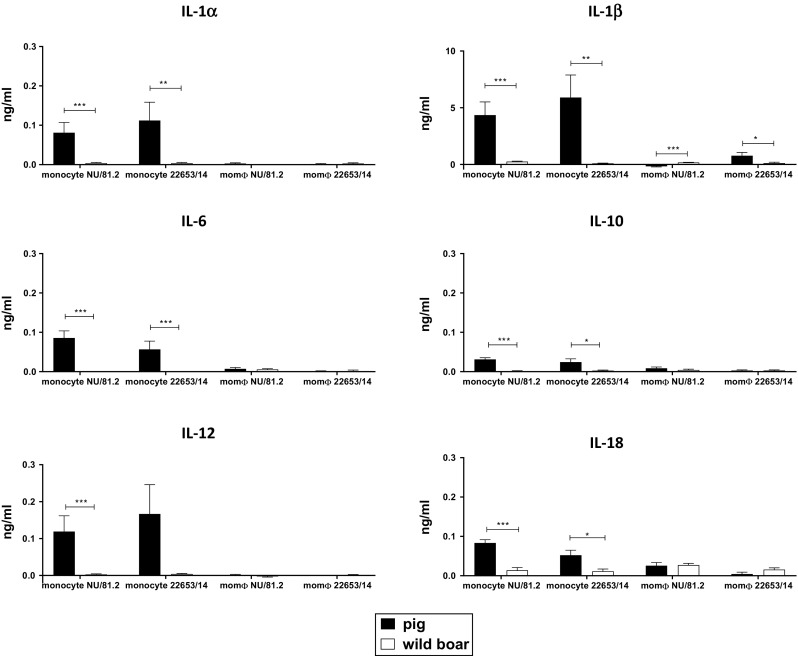
Release of cytokines by porcine and wild-boar monocytes and moMΦs in response to ASFV infection. Monocytes and moMΦs were infected with the virulent historical strain NU81.2 and the modern strain 22653/14 at an MOI of 1, alongside mock-infected controls. At 18 hours pi, the levels of GM-CSF, IL-1α, IL-1β, IL-6, IL-10, IL-12, and IL-18 in the culture supernatants were determined. Values were mock-corrected, and the mean data +/- SD from three independent experiments utilizing three different pigs and three different wild boars are shown. For each cytokine, for both monocytes and moMΦs, values from pigs and wild boars were compared using the Mann-Whitney test. ***, *p* < 0.001; **, *p* < 0.01; *, *p* < 0.05

Infection of pig monocytes with either modern or historical strains resulted in higher levels of IL-18, IL-1α, and IL-1β compared to the mock-infected control, although the increase in IL-1β was not statistically significant. Increased release of IL-1β in response to ASFV infection has been observed previously in other studies [6, 25], and here, we observed that there are no differences between historical and modern ASFV isolates. In addition, no differences between modern 22653/14 and historical NU81.2 were detected with respect to the levels of IL-18, IL-1α induced or in those of any of the other cytokines tested (Figure 3). Pig monocytes showed an increased production of IL-1α, IL-1β, IL-6, IL-12, IL-18 and IL-10 compared to pig macrophages and wild-boar cells following infection with both Sardinian ASFV strains. The highest amount of cytokine production was observed for IL1β (Figure 4), whereas IL-10 secretion appeared to be at the lowest level (0.04 ng/ml). Almost undetectable levels of TNF-α were detected (data not shown). In order to determine whether the higher cytokine levels found in domestic pigs compared to wild boars could be attributed to a different affinity of porcine and wild-boar cytokines for the antibodies used in the assay, we screened our samples using a specific ELISA kit (Wild boar IL1β ELISA kit, Antibodies-online.com), confirming the results obtained before (data not shown).

Several studies on the immune response to ASFV infection have been published, in particular, analysing the cytokine profiles of *in vitro*-cultured porcine monocytes/macrophages [6, 10–12, 25]. Comparative studies of virulent and attenuated ASFV strains have demonstrated a higher level of expression and production of relevant regulatory cytokines after infection with attenuated viruses [6, 10]. Having observed no differences between modern and historical Sardinian isolates, we suggest that they might both possess mechanisms to counteract monocyte/macrophage responses, promoting their survival and dissemination in the host.

## Conclusion

In conclusion, this study demonstrates that *in vitro* techniques used to differentiate pig monocytes into macrophages are also reliable when using a wild-boar model, allowing the use of this protocol in these animals as well. Modern Sardinian ASFV strains appeared to be more able to infect cells than the historical strain, although no differences were detected in the growth kinetics of strains 22653/14 and Nu81.2 in either monocytes or moMΦs, and no differences in the release of cytokines by monocyte or moMΦs in response to these isolates were detected. Using our *in vitro* model, we found evidence that wild boars and domestic pigs are equally susceptible to infection, even though the latter produce a stronger cytokine response. We are aware that our *in vitro* study provides only a partial view of the complex cell interactions that occur during natural infection, and further studies are needed, in particular, *in vivo* infection studies, in order to better understand the immune response mechanisms in domestic and wild pigs to ASFV infection.
